# Ubiquitously expressed Human Beta Defensin 1 (hBD1) forms bacteria-entrapping nets in a redox dependent mode of action

**DOI:** 10.1371/journal.ppat.1006261

**Published:** 2017-03-21

**Authors:** Judith Raschig, Daniela Mailänder-Sánchez, Anne Berscheid, Jürgen Berger, Adam A. Strömstedt, Lioba F. Courth, Nisar P. Malek, Heike Brötz-Oesterhelt, Jan Wehkamp

**Affiliations:** 1 Internal Medicine I, University Hospital Tuebingen, Tuebingen, Germany; 2 Department for Microbial Bioactive Compounds, Interfaculty Institute for Microbiology and Infection Medicine, University of Tuebingen, Tuebingen, Germany; 3 Max-Planck Institute for Developmental Biology, Electron Microscopy, Tuebingen, Germany; 4 Division of Pharmacognosy, Department of Medicinal Chemistry, Uppsala University, Biomedical Centre, Uppsala, Sweden; Stanford University School of Medicine, UNITED STATES

## Abstract

Ever since the discovery of endogenous host defense antimicrobial peptides it has been discussed how these evolutionary conserved molecules avoid to induce resistance and to remain effective. Human ß-defensin 1 (hBD1) is an ubiquitously expressed endogenous antimicrobial peptide that exhibits qualitatively distinct activities between its oxidized and reduced forms. Here, we explore these antimicrobial mechanisms. Surprisingly, using electron microscopy we detected a so far unknown net-like structure surrounding bacteria, which were treated with the reduced but not the oxidized form of hBD1. A transmigration assay demonstrated that hBD1-derived nets capture bacteria and inhibit bacterial transmigration independent of bacterial killing. The presence of nets could completely prevent migration of hBD1 resistant pathogens and are stable in the presence of human duodenal secretion with a high amount of proteases. In contrast to HD6, cysteins are necessary for net formation. This redox-dependent function serves as an additional mechanism of action for hBD1 and differs from net formation by other defensins such as Paneth cell-derived human α-defensin 6 (HD6). While hBD1red and hBD1ox have distinct antimicrobial profiles and functions, only the reduced form provides additional host protection by entrapping bacteria in extracellular net structures preventing bacterial invasion. Better understanding of the modes of action of endogenous host peptides will help to find new antimicrobial strategies.

## Author summary

The human body is constantly challenged by commensal and by pathogenic bacteria. Antimicrobial molecules of the innate immune system protect the human body from bacterial overgrowth and keep human microbiota in balance. One of the most prominent antimicrobial molecules is the human beta defensin 1 (hBD1). It is, in contrast to other defensins, constantly expressed by epithelial cells and exhibit diverse host antimicrobial strategies, which are much more complex than expected some years ago. Here, we aimed to study the unknown mechanisms of the different redox forms of hBD1. We detected, besides bacterial cell wall disruption, a so far unknown net-like structure surrounding bacteria, which entraps bacteria, independent of bacterial components and antimicrobial activity. Of note, these nets are depending on the amino acid cysteine and these nets are stable even in the presence of physiological human duodenal fluid. We demonstrated that net formation by reduced hBD1 prevents bacterial translocation, including hBD1 resistant pathogens. These findings highlight the complexity of these conserved antimicrobial defense mechanisms and help to find new antimicrobial strategies.

## Introduction

The innate immune system is the primary barrier against commensal invasion and microbial infections. Antimicrobial peptides (AMPs) are key effector molecules of the innate immune system protecting the human body from bacterial overgrowth, thereby retaining a balanced microbiota and fending off commensals and pathogens [1,2]. Some of the most important molecules within the innate immune system are the defensins. These are small cationic peptides of about 3 kDa characterized by three intramolecular disulphide-bridges [3–5]. According to the arrangement of these disulphide-bonds, defensins are classified as alpha- and beta-defensins, respectively [6]. Defensins show antimicrobial activity against various microbes, including bacteria, fungi and some viruses, whereby they shape the composition of the human microbiota [7]. While native human alpha-defensins 5 and 6 (HD5/ HD6) are expressed by Paneth cells in the small intestine and human beta-defensin 2 (hBD2) is mainly induced in case of inflammatory situations, human beta-defensin 1 (hBD1) is constitutively expressed by all human epithelia [8–10], indicating its important role in innate immunity. In a previous study, we could show that hBD1red is localized in human extracellular colonic mucus, suggesting that hBD1 can prevent infections *in vivo* [11]. However, the antimicrobial activity of hBD1 was previously undervalued. By using reducing conditions, as they are found in the human intestine, we could show antimicrobial activity of hBD1 against numerous members of the human intestinal microbiota [12] as well as against several pathogenic bacteria. In contrast, oxidized hBD1 (hBD1ox), which contains three closed disulfide bonds, showed no antimicrobial activity against most microbes, with the exception of a few Gram-negative bacteria including *E*. *coli* [12]. It is known that different AMPs have different bacterial target sites or mechanisms of activity, as recently reviewed by Brogden [13]. For example, *Sass et al*. showed that human ß-defensin 3 kills *S*. *aureus* by interfering with membrane-bound multienzyme-machineries such as the electron transport chain and the cell wall biosynthesis complex [14]. Chileveru et al. described a different mechanism for human Paneth cell alpha-defensin 5 (HD5) [15]. They reported that HD5 enters the cytoplasm of *E*. *coli* and other Gram-negative bacteria, leading to morphological changes like blebbing, clumping and cell elongation [15], followed by the loss of bacterial viability. Paneth cell HD6 was reported to form net-like structures, entrapping *S*. *typhimurium*, thereby preventing the translocation of these bacteria into the intestinal epithelium [16].

Similar to hBD1, Paneth cell HD6 also shows strong antimicrobial activity after the reduction of its disulphide bonds [17]. The same basic principle of broadened activity is also true for Psoriasin, a S100 protein with strong presence especially in the skin [18]. However, so far the majority of literature suggests a single mechanism and spectrum for each host defense peptide, respectively. This notion of one mechanism of action per antimicrobial peptide is typically a result of experimental design of most studies, which are generally based on environmental conditions that do not reflect *in vivo* conditions.

Based on the wide expression at all sites of the body, we focused our current research on a better understanding of hBD1. Previous data from our group suggested no differences in terms of killing of Gram-negative *E*. *coli*, which was affected by both, the reduced and oxidized form of hBD1 [12]. After performing different distinct experimental approaches, we found here that this preliminary observation did not show the full picture and the mechanism of affecting Gram-negative *E*. *coli*, which differs dramatically between the different forms of hBD1. While we show here that hBD1red is active in Gram-positive and Gram-negative strains by targeting mainly the bacterial membrane, hBD1ox seems to act only in Gram-negative bacteria, without a significant effect on the cell envelope. In particular, we aimed to elucidate the mechanisms by which hBD1red inhibits bacterial growth. To our surprise, electron microscopic analyses of hBD1red-treated bacteria revealed a net-like structure which could not be seen with the oxidized form of the same peptide. In this work we show at different levels that the same peptide provides distinct and complementary killing-based and killing-independent antimicrobial mechanisms supporting its diverse and important role in shaping the microbiota.

## Results

### The two redox forms of hBD1 exhibit different anti-bacterial mechanisms

**Fig 1:**
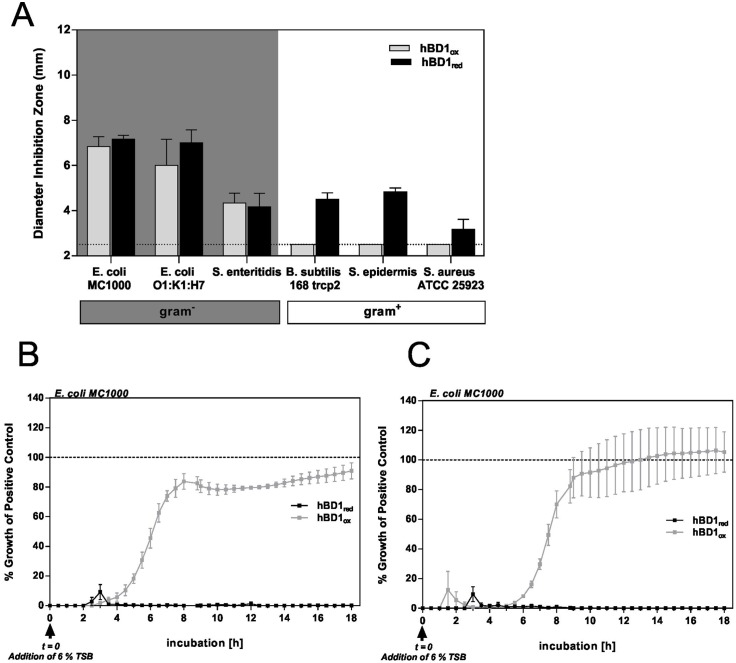
Antimicrobial activity of hBD1 is dependent of its redox status. Bacteria were incubated with hBD1ox and hBD1red in (A) Radial Diffusion Assay. Antimicrobial activity was measured by analyzing the diameter of the inhibition zone. A diameter of 2.5 mm (dotted line) is the diameter of the punched well (n = 3). As it is not possible to distinguish between a bactericidal and a bacteriostatic mechanism using RDA as an end-point determination, we additionally performed a time-course in a turbidity broth assay. Bacteria were pretreated with 62.5 μM (B) and 125 μM (C) of the peptides before addition of 6% TSB and % growth of the untreated control over the following 18 h was plotted. In contrast to hBD1red, the *E*. *coli* culture treated with hBD1ox is able to regrow with a delay of 4 h (B) and 5.5 h (C) compared to the control. Data are presented as mean +/- SEM of at least two (B+C) or three (A) independent experiments.

We reported previously that hBD1 can exhibit broad antimicrobial activity under reducing conditions, while only *E*. *coli* was affected by the oxidized form of hBD1 (hBD1ox) [12]. In this study, we used synthetically produced hBD1red, instead of subjecting hBD1 to reducing conditions, e.g. by addition of DTT. Therefore, we aimed to confirm the antimicrobial activity of synthetically produced hBD1red using radial diffusion assays (RDA). In line with our previous data [12], hBD1ox showed antimicrobial activity against Gram-negative *E*. *coli* strains and the tested *S*. *enteritidis* but not against Gram-positive *B*. *subtilis* and *S*. *aureus* strains while hBD1red was strongly active against all tested bacterial strains, including Gram-positive bacteria (Fig 1A). As it is not possible to distinguish between a bactericidal and a bacteriostatic mechanism using RDA as an end-point determination, we additionally performed a time-course in a turbidity broth assay. As shown in Fig 1B and 1C, hBD1red completely inhibited *E*. *coli* growth at concentrations of 62.5 μM (Fig 1B) and 125 μM (Fig 1C) while hBD1ox only delayed growth of *E*. *coli* in comparison to the untreated control, even at the higher dose. Our data indicate a bactericidal mode of action for hBD1red.

**Fig 2:**
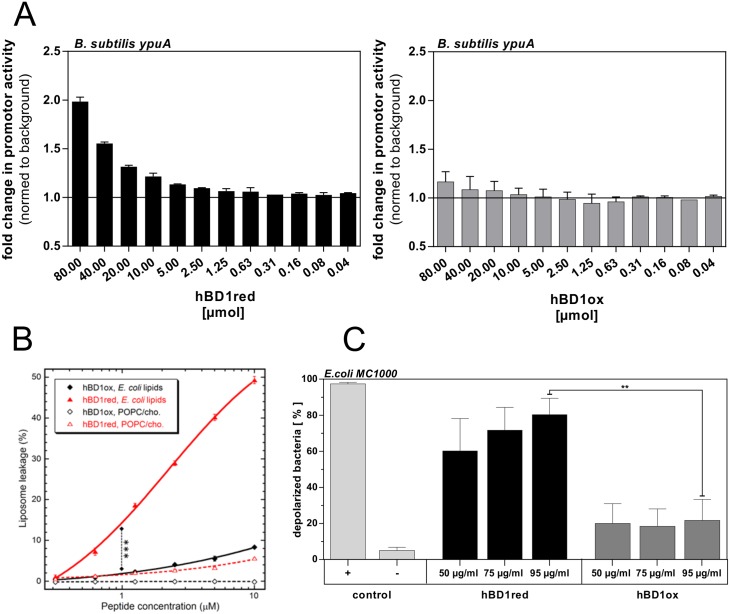
Characterization of the hBD1red target in bacterial compartments. (A) Transcription activity of the *B*. *subtilis ypuA* promoter indicative of cell envelope damage. Bacteria were incubated with increasing concentrations of hBD1ox and hBD1red. (B) Membrane leakage as a result of hBD1 treatment was determined by quantifying carboxyfluorescein efflux from liposomes POPC/cholesterol (3:2 molar ratio, a human membrane model) or an *E*. *coli* polar lipid extract. Presented is the leakage level reached after 45 min incubation with mean and standard deviations from triplicates. Permeabilization, by hBD1red on *E*. *coli* liposomes, was significantly higher than by hBD1ox (***p<0.001 only for 1 μM). (C) Membrane depolarization of 1.5 x 10^6^ CFU *E*. *coli* MC1000 in response to hBD1. 1 h treatment was analyzed by flow cytometry. Positive control (+) was incubated with hBD3ox (50 μg/ml) and the negative control (-) without any peptide. The statistic was evaluated by using student’s t-test with **p = 0.0072. Data are presented as mean +/- SEM of at least three independent experiments.

Antimicrobial peptides frequently act at the bacterial cell membrane, leading to pore formation, breakdown of membrane potential and sudden bacterial cell death [5]. Other AMPs target cell wall synthesis and other envelope related factors [19]. To clarify whether hBD1ox and hBD1red, respectively, also target the bacterial cell envelope, we used a bacterial reporter strain of *B*. *subtilis*, which carries the *ypuA* promoter fused to the firefly luciferase reporter gene. The *ypuA* promoter was shown to be sensitive to membrane-associated and cell wall related stress [20]. Therefore, increased luciferase activity indicates cell envelope impairment. We modified the standard protocol from Wenzel et al, for using and testing antimicrobial peptides [21] and probed the *ypuA*-reporter strain with increasing concentrations of hBD1ox and hBD1red. As shown in Fig 2A, the *ypuA* promoter was significantly activated (two fold) by hBD1red, whereas no activation was observed for hBD1ox, in the concentration range tested. As *B*. *subtilis* was not sensitive to hBD1ox in the RDA, the significance of this assay was very limited with regard to hBD1ox. Therefore, we aimed to strengthen our results by additional methods using Gram-negative bacteria, which are sensitive to both forms. In a membrane permeabilization assay we used liposomes generated from either an *E*. *coli* polar lipid extract or model membranes, intended to mimic a human cytoplasmic membrane, composed of POPC:cholesterol (3:2). Permeabilization was monitored via the fluorescent signal generated by efflux of an encapsulated self-quenching marker, in response to different hBD1ox and hBD1red concentrations. HBD1ox, after 45 minutes at the highest concentration tested (10 μM), caused only 8% leakage from the bacterial liposomes and no leakage at all from the human liposomes (Fig 2B). HBD1red, on the other hand, caused about 8% leakage already at 0.6 μM and reached its EC50 at 10 μM with the bacterial liposomes. The leakage kinetic was fast with the majority of the leakage generated within the first 10 minutes after which a leakage plateau was reached, representing a kinetic profile typical of membrane active AMPs. On the POPC/cholesterol membrane the reduced form lead only to 6% leakage at 10 μM. This represents a very low level of “self-toxicity”, especially since this type of artificial lipid compositions is substantially more susceptible to peptide induced leakage compared to natural lipid compositions [22]. In terms of phospholipid composition, the *E*. *coli* liposomes can be considered a Gram-negative membrane model, and the activity reached with hBD1red on these liposomes is relevant for bactericidal properties observed in other assays used in this study. These results point towards the cell membrane as one target for hBD1red and are in line with the results obtained using the *B*. *subtilis* reporter strain. However, hBD1ox displayed only a weak impact on the bacterial liposomes. To further assess a possible effect of hBD1red against bacterial cytoplasmic membrane we used a flow cytometric assay, which detects membrane depolarization [23]. To this end, *E*. *coli* bacteria were incubated with increasing concentrations of hBD1ox and hBD1red (Fig 2C). We observed a high percentage of depolarized bacteria after 1 h of treatment with hBD1red. Approximately 80% of the bacteria displayed depolarized membranes. In contrast, after incubation with hBD1ox only 20% of the bacteria were depolarized, whereas the negative control exhibited 7% spontaneous depolarization events. Taken together, the results from these three different assays demonstrate that hBD1red kills bacteria by broadly targeting bacterial membranes, while the antibacterial effect of hBD1ox is much more species specific.

### Bacteria are entrapped in a net-structure formed by hBD1red

**Fig 3:**
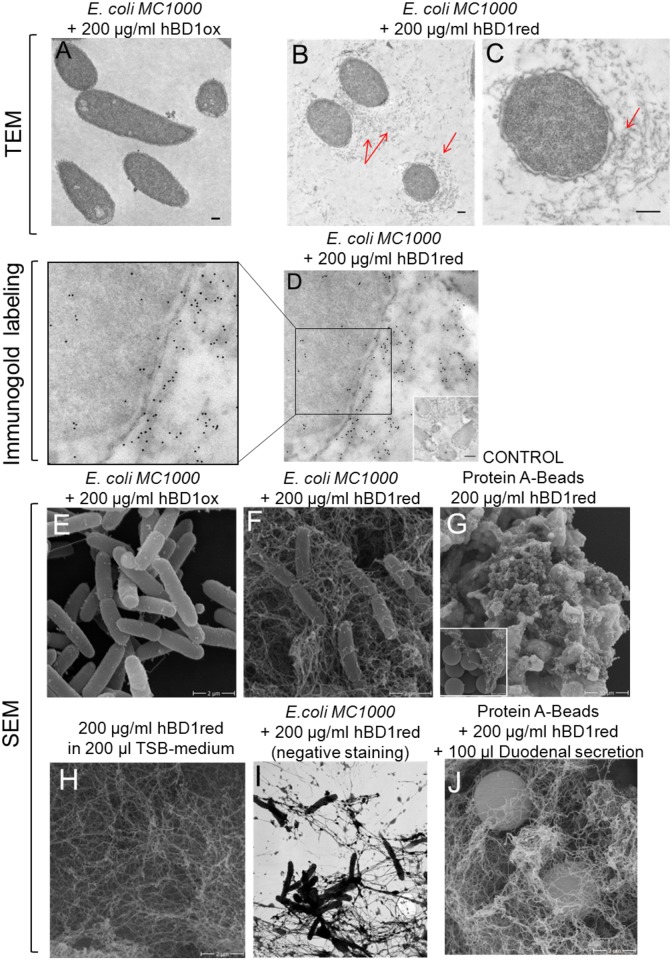
HBD1red forms netlike structures. Bacteria or beads were treated with hBD1 redox forms for 2 h. Samples were fixed and visualized by electron microscopy. (A-C) Transmission electron microscopy of *E*. *coli* MC1000 for analyzing the bacterial morphology. HBD1red-treated *E*. *coli* cells display strong membrane defects and an unknown grey structure around the bacteria was detected. (D) *E*. *coli* MC1000 was incubated with hBD1red and with antibodies against the peptide. The secondary antibodies are conjugated to gold particles, 6 nm (black points). (E) *E*. *coli* MC1000 were treated with hBD1ox. (F-I) Bacteria or protein A-coated beads were treated with hBD1red and investigated by scanning electron microscopy. (G) As a control we used hBD1red with beads (H) and alone in medium without bacteria and beads. (I) HBD1red net formation in native samples without addition of fixative agents. (J) Protein A-coated beads were incubated with hBD1red and human duodenal secretion, which was taken from one individual.

To further investigate the different killing mechanisms of hBD1ox and hBD1red, we used electron microscopy to visualize peptide treated bacteria (control bacteria; S1 Fig). First, we analyzed changes in bacterial morphology in response to hBD1ox and hBD1red, respectively. In agreement with the membrane assays, hBD1ox did not cause any visible damage to the bacterial cell wall or membrane, nor did we see any cytosolic damage (Fig 3A). In contrast, hBD1red led to strong cell envelope damage in *E*. *coli* and the cell membrane was detached from the cell wall. Interestingly, a dense structure around the bacteria after hBD1red treatment was observed (Fig 3B + 3C, arrows). This structure was localized near the outer membrane and parts of the outer membrane were entangled with the surrounding meshwork. To further analyze this structure, we studied the localization of hBD1red using immuno-gold labeling (controls; S3 Fig). We detected high amounts of hBD1red all around the bacterial cells and, to a lesser extent, in the cytosol (Fig 3D). Interestingly, the dense structure around the bacteria seemed to be composed of hBD1red. To confirm this, hBD1red was incubated with *E*. *coli* and visualized by scanning electron microscopy (SEM). SEM displayed the unknown structure as a meshwork of an extracellular net surrounding the bacteria. These data indicate that hBD1red is able to form nets, which can entrap bacteria as seen in Fig 3F. To clarify whether this net-like structure is generated by hBD1red or by bacterial components, experiments independent of bacteria were performed. Therefore, protein-A coated beads were incubated with hBD1red (Fig 3G). We observed that hBD1red, with and without beads, was capable of forming net-like structures independently of microbes (Fig 3H). This finding is in contrast to Paneth cell HD6 that only forms nets in the presence of bacterial structures [16], such as flagella and type I fimbria. To exclude any unspecific reactions, e. g. with the Karnovsky’s reagents, we analyzed the net formation of hBD1red with bacteria in absence of any fixative (Fig 3I). Additional the net formation was also stable in presence of human duodenal fluid, which contains a high amount of human proteases (Fig 3J). This observation suggests that the net structure is resistant against physiological occurring proteases and does occur under physiological conditions, even if the experiments were performed *ex vivo*. Again, we observed formation of nets, strengthening our hypothesis of net-formation as an additional mechanism in hBD1red antimicrobial activity. In order to test if the thioredoxin (TRX) system is able to trigger net formation, we incubated this redox enzyme with hBD1ox. As expected, the TRX system transforms oxidized hBD1 into the reduced peptide which forms nets (S2 Fig). In contrast to hBD1ox, only reduced hBD1 entraps bacteria, which was completely unknown until now.

### Cysteines are important for net-formation of hBD1red

**Fig 4:**
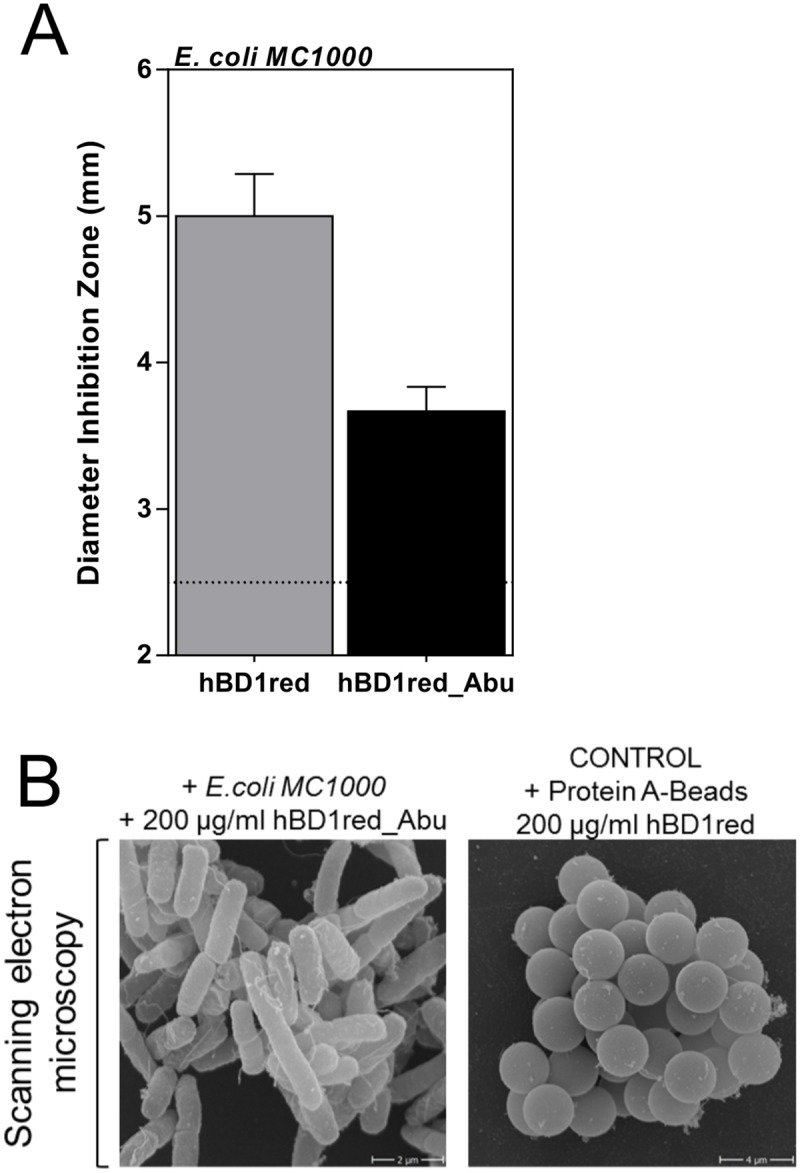
Net-formation of hBD1red depends on free cysteines. (A) 2 μg of hBD1red and hBD1red_Abu, a variant in which cysteine is replaced by α-amino-butyric acid (Abu), were tested in a RDA Assay against *E*. *coli* MC1000 or (B) 200 μg/ ml peptide with bacteria or protein A-coated beads were analyzed by scanning electron microscopy. Data in A) are presented as mean +/- SEM of at least three independent experiments.

We previously described the formation of nets by HD6, as well as its antimicrobial activity, to be independent of cysteines [17]. We therefore investigated if this was also true for hBD1red. Consequently, all cysteines were replaced by α-aminobutyric acid (Abu), a synthetic analog of cysteine, with the thiol group on the sidechain exchanged for a methyl group. The resulting modified peptide (hBD1red_Abu) was tested for its antimicrobial activity in RDA. As shown in Fig 4A, hBD1red_Abu showed lower antimicrobial activity compared to the native hBD1red. Furthermore, hBD1red_Abu was not able to form nets, neither with bacteria, nor with protein-A beads (Fig 4B). We therefore conclude that the antimicrobial activity and net-formation of hBD1red is dependent on free cysteines in the reduced peptide. Importantly, the six Cys-to-Abu substitutions represent an increase in hydrophobicity of the peptide which normally results in an increase of potency for membrane active AMPs, particularly in terms of indiscriminate lytic activity [24].

### HBD1red-nets entrap bacteria and inhibit translocation

**Fig 5:**
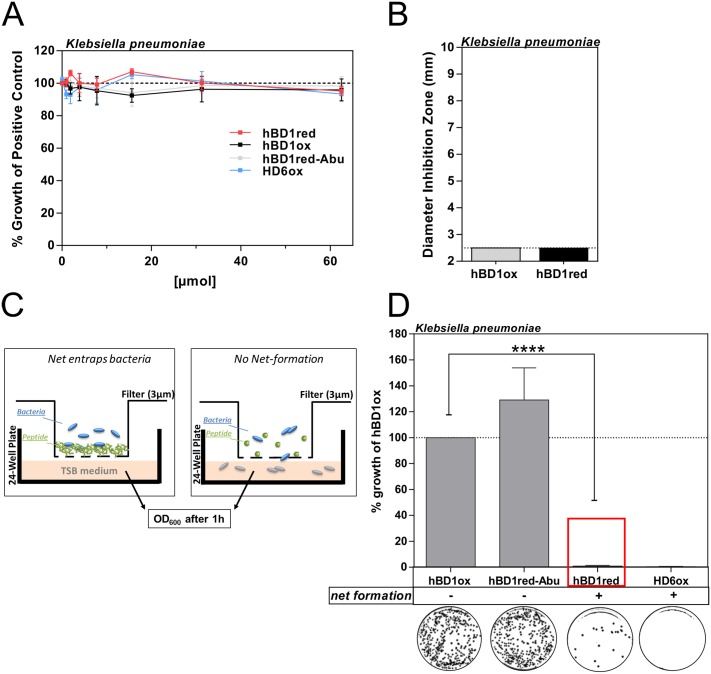
Proof for functional net-formation of hBD1red. Comparison of antimicrobial activity of different hBD1 forms. Antimicrobial activity of hBD1 forms was compared in a (A) turbidity broth assay or (B) RDA against *K*. *pneumoniae*. Different concentrations of peptides were incubated with bacteria and change in optical density (OD_600_) was measured after 12 h or the diameter of inhibition zone was analyzed. HBD1 redox forms are not antimicrobial active against *K*. *pneumoniae*. (C) Schematic model for the Transwell Membrane Assay. Bacteria are entrapped in a net and are not able to pass the porous membrane (left), but in absence of nets bacteria pass through the membrane and grow in the TSB medium in the chamber below. (D) Bacterial growth was measured at OD_600_ after 1 h pre-incubation of peptide in filter and additional 2 h and % growth of negative control was plotted. With net formation no significant numbers of bacteria are able to translocate which is in contrast to hBD1ox or hBD1red_Abu with no net formation. Representative blood agar plates are shown. Data are presented as mean +/- SEM of at least three independent experiments. The statistic was evaluated by using student’s t-test with ****p < 0.0001.

To test whether hBD1red-nets are able to inhibit bacterial translocation functionally, we established a transmigration assay using a filter trans-well system (Fig 5C). To exclude false-positive results due to bacterial killing by hBD1, we selected a bacterium, which is resistant against hBD1ox and hBD1red. As shown in Fig 5A and 5B, *K*. *pneumoniae* is resistant to hBD1ox, hBD1red, hBD1red_Abu and HD6, respectively, up to concentrations of 62.5 μM. We therefore used *K*. *pneumoniae* to show the functionality of hBD1red-nets. For that purpose, we prepared trans-well filters (3 μm pore size) with hBD1ox, hBD1red_Abu and hBD1red. Additionally, we used HD6 as a positive control, as the functionality of HD6-nets in preventing bacterial translocation has already been shown *in vivo* [16]. Next, suspensions of *K*. *pneumoniae* were added to the prepared filters. Peptides able to form functional nets would prevent *K*. *pneumoniae* from transmigrating to the lower compartment of the trans-well system (Fig 5C). *K*. *pneumoniae* was incubated in the trans-well chamber for 1 h and bacterial growth in the lower compartment was assessed by measuring OD_600_. Additionally, 10 μl medium taken from the lower compartment were plated on blood agar plates (Fig 5D). When filters were incubated with hBD1ox and hBD1red_Abu, we found strong bacterial growth in the lower compartment, which shows that bacteria are not restrained and can translocate through the filter. This is in line with our previous results showing incapability of hBD1ox and hBD1red_Abu for net-formation. However, when preparing the filter with hBD1red and HD6ox (Fig 5D), we did not detect any bacterial growth in the lower compartment. We therefore conclude that hBD1red forms functional nets that trap bacteria, thereby preventing their translocation without necessarily killing the microbes.

## Discussion

If a conventional antibiotic is used repeatedly, microbial resistance against this antibiotic is the rule rather than the exception. Multiresistant bacteria are increasing and more and more become a serious threat for our world in the modern antibiotic era [25,26]. Ever since the discovery of endogenous host defense antimicrobial peptides it has been discussed why these evolutionary conserved molecules do not evoke resistance, especially in a constant use situation. For a long time after the discovery of hBD1, scientists wondered why an antimicrobial peptide exhibits so little antimicrobial activity. HBD1 is likely one of the most abundant known antimicrobial peptides because it is constantly expressed not only by all epithelial surfaces but also by circulatory cells and cells of the reproductive tract. Polymorphisms of hBD1 are associated with an inefficiency to clear potential harmfully microbes like MRSA [27], and various diseases like periodontitis [28], psoriasis [29], colonic inflammatory bowel diseases [30,31] and infertility [32]. Recently, we have shown that hBD1 becomes a potent AMP after reduction of its disulphide bonds [12]. Furthermore, we could show that this reduction can be performed by the thioredoxin system, which is present in the human intestine, where it co-localizes with hBD1red [11]. The data provided here demonstrate additional fundamental principles of the permanent war at mucosal surfaces where the host has to be protected against pathogens as well commensal overpopulation [33]. These data highlight the contrast between most conventional antibiotics, primarily of bacterial origin, and human endogenous host defense molecules such as hBD1, the latter providing multifunctional activities, including distinct antimicrobial mechanisms from a single peptide. Herein, we show that, post-translationally, hBD1 forms functionally distinct structures, which are controlled by the local microenvironment. Our data show that hBD1ox is only active against some Gram-negative species (*S*. *enteritidis*, *E*. *coli)* in (solid) radial diffusion assays (Fig 1A). Interestingly, *E*. *coli* showed decreased sensitivity to hBD1ox in a turbidity (liquid) broth assay. While hBD1red was bactericidal and sterilized the culture, viable cells remained in the presence of hBD1ox and the treated culture eventually reached the same turbidity as the untreated control (Fig 1B + 1C).

The bacterial cell envelope is a commonly discussed target for AMPs [19] and cell envelope impairment has been described as a specific mechanism of defensins [5]. Using a *B*. *subtilis* reporter strain, we were able to show that hBD1red causes cell envelope stress in this organism, while hBD1ox does not (Fig 2A). Using this assay, we got first indications for the bacterial target site of hBD1red. As *B*. *subtilis* is not sensitive to hBD1ox, we further confirmed these findings, using hBD1ox sensitive *E*. *coli*, by analyzing the membrane as a target in two different assays. Both methods revealed a loss in membrane integrity (Fig 2B) or breakdown of membrane potential (Fig 2C) due to hBD1red, while hBD1ox was not able to significantly affect the bacterial membrane. Our findings illustrate that bacterial membrane disruption is involved in the direct antibacterial activity of the reduced hBD1, only. Herein, we displayed differences between hBD1ox and hBD1red, thus our work highlights the importance that one peptide can have diverse killing strategies, depending on the environmental settings. To analyze morphological changes in *E*. *coli* treated with hBD1, TEM imaging was performed. Again, this method revealed marked differences between hBD1ox and hBD1red. HBD1red treated *E*. *coli* showed signs of cell membrane damages, confirming our first results (Fig 3B + 3C). Morphological changes in *E*. *coli* in response to the AMP HD5 have recently been described by Chileveru et al. They found cell elongation and clumping in *E*. *coli* after treatment with HD5ox. In contrast, hBD1 did not lead to cell elongation or clumping in our experiments. In addition to cell envelope damages described above, we found a net-like structure surrounding *E*. *coli* treated with hBD1red. Other studies aiming at localizing AMPs within their microbial targets used AMPs labeled with fluorescence markers [15,34]. Due to the small size of AMPs, it cannot be excluded that tagging with a fluorophor would alter the antimicrobial activity. Therefore, we used specific antibodies and immunogold labeling to localize hBD1red, instead. We detected large amounts of hBD1red, especially the aforementioned net-like structures, surrounding the *E*. *coli* cells, staining highly positive for hBD1red (negative controls in S4 Fig). We therefore hypothesized that these nets could be comprised of hBD1red, similar to the nets formed by HD6 [16]. To test this hypothesis, we performed SEM analysis. SEM confirmed the presence of a net-like structure after treatment with hBD1red. Furthermore, by using protein A beads and a control containing only hBD1red, we could show that only the presence of hBD1red is required for net formation, strengthening our hypothesis that this so far undescribed net-like structure is indeed comprised by hBD1red molecules. Of note, this net structure also occurs in a physiological environment, such as duodenal fluid, which also contains high levels of proteases (S5 Fig). One could think that net forming structures are similar between different defensins, like HD6 and hBD1, but this does not seem to be the case. In contrast to HD6, for which Chu et al described the formation of nets to be dependent of the presence of bacterial components, this is different for hBD1red. Also, while both redox forms of HD6 are able to form nets [17], only the reduced form of hBD1 shows this ability. HBD1 has 6 cysteines that form 3 disulphide bridges, characteristic for defensins, under oxidizing conditions. In reduced hBD1, these disulphide bridges are open, creating free sulfhydryl groups that could provide the origin for intermolecular links. To test this hypothesis we replaced hBD1red cysteines by α-amino butyric acid (hBD1red_Abu). HBD1red_Abu was no longer able to form nets, indicating the importance of free cysteines for net-formation. That the hBD1red_Abu exhibited less bioactivity as compared to hBD1red shows unambiguously that there is more to the hBD1 redox activity difference than mere hydrophobicity. Again, this is in contrast to HD6red where net formation was independent from free cysteines [17]. To demonstrate the ability of hBD1red nets to prevent bacterial translocation, we designed a migration assay using a transwell filter membrane and a bacterium resistant to hBD1red, *K*. *pneumoniae*. First, we applied a pre-incubation time of more than 10 min to ensure a stable net building of reduced hBD1 (S4 Fig). Nets of hBD1red markedly reduced migration of *K*. *pneumoniae* from the upper to the lower compartment of the transwell system. The same was true for HD6 nets, which served as a positive control, even the mechanisms are different, as described above. Again, our findings are in line with *Chu et al* who could show the relevance for HD6 nets *in vivo*, using a transgenic HD6 mouse. In this model, HD6 nets prevented translocation of *S*. *typhimurium* into the mouse ileal tissue. Of note, the expression profile of hBD1 is much broader, suggesting a more general defense principle at different surfaces. Taken together, our results suggest an additional, so far unknown mechanism of hBD1red, beyond direct killing of bacteria. The reduced but not oxidized form of hBD1 forms nets, entrapping bacteria and preventing bacterial translocation independent of bacterial killing. Of note, this mechanism is distinct from the described HD6 net formation, indicating the broad spectrum of evolutionarily conserved resistance mechanisms. We could previously show the presence of hBD1red in the mucus of human colonic tissue [35]. In addition to the antimicrobial activity of hBD1red the formation of nets in colonic mucus provides a further barrier for commensals and pathogens. Despite the similarities between HD6 and hBD1, there are also marked differences, not only between hBD1 and HD6, but also between hBD1ox and hBD1red, thus our work again highlights the importance of considering host defense peptides individually and in the context of the appropriate environmental conditions, instead of seeking a general killing mechanism. The so far unique multitasking capacities of this broadly expressed host defense peptide could explain it’s primarily role in different diseases, like periodontitis, infertility, susceptibility to MRSA infections and chronic inflammatory diseases, such as chronic inflammatory bowel diseases, all associated with defective hBD1 production. The “buy one get much more strategy” of surface defense might also explain why the oldest evolutionary battle between eukaryotes and microbial threat is not lost despite many million years of permanent exposition. These distinct activities of hBD1, as shown here, represent an excellent example of how a host defense peptide can utilize multiple strategies to combat bacterial challenges directly.

## Materials and methods

### Microbial strains

Bacterial strains *E*. *coli* (O1:K1:H7) DSM 10729 and *Staphylococcus epidermidis* DSM 20044 were obtained from the Deutsche Sammlung von Mikroorganismen und Zellkultur GmbH (Braunschweig). *Salmonella enteritidis*, *Staphylococcus aureus* ATCC 25923, *Klebsiella pneumoniae* 3-MRGN were provided by the Department for Laboratory Medicine at Robert-Bosch-Hospital Stuttgart. *E*. *coli* MC1000 strain was kindly provided by Jean-Francois Collet (Belgium, Brussels, Universite Catholique de Louvain). *Bacillus subtilis* 168 (*trpC2*) was obtained from the Interfaculty Institute for Microbiology and Infection Medicine, Tuebingen, Germany.

### Human physiological duodenal fluid

Human duodenal secretion was obtained from one representative healthy individual during routine duodenoscopy. Informed consent was obtained in all cases prior and the local ethical committee of the University of Tübingen approved collecting human material for this study.

### Proteolytic activity of human duodenal secretion

Proteolytic activity was assayed by using 2.5% (w/v) azocasein solution (Sigma). Briefly, 2 ml of human duodenal secretion from a representative healthy individual or 10 μl of trypsin enzyme solutions (10 mg/ml) (positive control) or without enzyme solutions (negative control) were added in reaction buffer (50 mM ammonium bicarbonate buffer, pH 7.8, 37°C). The mixture was incubated at 37°C for 30 min. The reactions were terminated by adding 800 μl of 5.0% (v/v) trichloracetic acid, followed by centrifugation at 14.000 rpm for 5 min. 100 μl of the supernatant was neutralized by adding 100 μl of 500 mM NaOH and the absorbance at 440 nm was measured. Experiment was repeated using independent experiments. Data are presented as mean +/- SEM of two experiments.

### Ethics statement

The study protocol was previously approved by the ethical committee of the University Hospital Tuebingen, Germany. Patients and controls who were included in this study all gave their written and informed consent after the study purpose, sample procedure, and potential adjunctive risks were explained.

### Peptides

All reduced peptides were chemically synthesized by EMC Microcollections (Tuebingen, Germany). The oxidized peptides were obtained from Peptide Institute (Japan).

### Membrane permeabilization assay

The liposome preparation and leakage assays were performed as described in detail by Strömstedt et *al*., with modifications necessitated by specific lipid compositions [22]. In brief, dry lipid films of either *E*. *coli* polar lipid extract or 1-palmitoyl-2-oleoyl-sn-glycero-3-phosphatidylcholine (POPC), with cholesterol (POP:cho in 3:2 molar ration, Avanti Polar Lipids, Alabaster, US-AL), were formed by dissolving lipids in chloroform, followed by evaporation in round bottom glass vials. The lipid films were re-suspended either by 30 minutes stirring (*E*. *coli*), or repeated freeze-thawing with liquid nitrogen (POPC:cho), at 55°C in an aqueous solution of 100 mM 5(6)-carboxyfluorescein. Suspensions were repeatedly extruded through 100 nm polycarbonate membranes in order to reduce multilamellar structures and polydispersity. Untrapped carboxyfluorescein was removed by gel filtration on Sephadex PD-10 columns. Membrane permeability was measured by monitoring carboxyfluorescein efflux from the liposomes, resulting in loss of self-quenching and an increased fluorescence signal. Fluorescence was monitored in a 96-well plate format with a peptide serial dilution to which liposome solutions were auto-dispensed. Induced leakage was monitored for 45 min in 10 mM Tris buffer (pH 7.4, 37°C) in triplicate, using the detergent Triton X-100 to define maximal leakage. The statistic was evaluated by using t-test with *** P < 0.001.

### Radial Diffusion Assay (RDA)

RDA was performed as described earlier [36]. Briefly, log-phase bacteria were grown in liquid tryptic soy broth (TSB, Becton Dickinson, USA) and washed with 10 mM sodium phosphate buffer; pH 7.4. Anaerobic bacteria were cultivated using AnaeroGen pouches (Oxoid, UK) to generate an anaerobic atmosphere. Bacteria were adjusted to 4 x 10^6^ CFU/ ml in 10 mM sodium phosphate buffer, pH 7.4 with 0.3 mg / ml TSB powder and 1% (w/v) low EEO-agarose (AppliChem). Bacteria were incubated under anaerobic or aerobic conditions, respectively, with 2 or 4 μg of synthetic, oxidized hBD1 and 2 or 4 μg synthetic, reduced hBD1 for three hours. Plates were then covered with 10 ml sterile liquid agar, containing 6% (w/v) TSB powder, 1% (w/v) agar and 10 mM sodium phosphate buffer (“overlay-gel”) and incubated for 16 h at 37°C. The diameter of the inhibition zones corresponds to the antimicrobial activity, but a diameter of 2.5 mm is the diameter of the punched well. Experiments were repeated at least three times; mean +/- SEM is shown.

### Turbidity broth assay

Log-phase bacteria were washed twice with 10 mM sodium phosphate buffer, containing 1% (w/v) TSB and the optical density at 600 nm (OD_600nm_) was determined. We used 5 x 10^5^ CFU/ ml, which were incubated with serial peptide concentrations (0.97–62.5 μmol) in a final volume of 100 μl of 10 mM sodium phosphate buffer containing 1% TSB for 2 h at 37°C. After incubation, 100 μl of 6% TSB (w/v) were added and optical density was measured at 600 nm for 18 h (BioTek Plate reader Synergy HT). Growth relative to the positive control in % was plotted against peptide concentration and experiments were repeated at least two times; mean +/- SEM is shown.

### Luciferase reporter gene assay

We used a specific bacterial reporter strain for a quantitative expression analysis of a selected marker gene [20,21]. The strain, with the genetic background of *B*. *subtilis* 1S34, carried the promoter *ypuA*, which is fused to the firefly luciferase reporter gene. Bacteria were grown at 37°C in lysogeny broth (LB) with 5 μg/ml erythromycin (resistance marker of the luciferase plasmid) to an OD_600_ of 0.9 and diluted to an OD_600_ of 0.02. Serial peptide dilutions (0.039–80 μmol) in 60 μl LB medium were prepared in a microtiter plate and were incubated with 60 μl of the adjusted bacterial suspension at 37°C for 1h. Then 60 μl citrate buffer (0.1M, pH 5), containing 2 mM luciferin (Iris Biotech, Germany) was added and luminescence was measured using a microtiter plate reader (Tecan, Switzerland).

### Flow cytometry assay

To get hints for membrane damages we used approximately 1.5 x 10^6^ CFU log-phase bacteria in a final volume of 95 μl TSB (1:6 diluted in H2O). We added peptides in concentrations 50, 75 and 95 μg/ ml in a final volume of 10 μl and incubated these suspensions for 1h at 37°C. Subsequently, 1 μg/ml of the membrane potential sensitive dye DiBAC_4_(3) [bis-[1,3-dibutylbarbituric acid) trimethine oxonol] (Thermo Fisher Scientific, USA) was added and incubated for 10 min at room temperature. Then, bacteria were centrifuged (5 min at 4°C and 7000 rpm) and re-suspended in 300 μl PBS. The percentage of depolarized fluorescent bacteria was determined using a FACSCalibur flow cytometer (BD, Sparks, USA), as described earlier [23]. Experiments were repeated at least three times and mean +/- SEM is shown.

### Scanning electron microscopy of bacteria

For scanning electron microscopy (SEM) approximately 1.2 x 10^9^ CFU/ ml of *E*. *coli* were incubated in 10 mM sodium phosphate buffer containing 1% TSB with 200 μg/ ml peptide for 2 hours at 37°C. After centrifugation at 7000 rpm, 5 min, and 4°C, the bacterial pellet was fixed in Karnovsky’s reagents. Bacteria were washed in PBS and finally fixed with 1% OsO_4_ in H_2_O. Samples were dehydrated to 100% ethanol and critical-point dried with CO_2_. Analysis was performed by a Hitachi S-800 field emission scanning electron microscope (Tokio, Japan) in Tuebingen (Max Planck Institute of Developmental Biology, Tuebingen, Germany).

### Immunogold labeling/ Transmission electron microscopy

Transmission electron microscopy for morphologic analysis of bacteria was performed as previously described [37]. For immunogold-hBD1red staining 1.2 x 10^9^ CFU/ ml of *E*. *coli* were incubated with 200 μg/ ml peptide for 2 hours at 37°C. Bacteria were centrifuged and the pellet was fixed with 3.0% paraformaldehyde, 0.01% glutaraldehyde. The next step was an additional centrifugation and the resulting pellet was embedded in 4% agarose at 37°C and then cooled down to room temperature. Small parts of agarose blocks were embedded in Lowicryl K4M (Polysciences, Germany). Ultrathin sections were cut using an ultramicrotome (Ultracut; Reichert, Vienna Austria). For immunogold labeling the ultra-thin sections (30 nm) were mounted on formvar-coated nickel grids and incubated with rabbit-anti-hBD1red, which was generated in reference [12]. Finally, the samples were incubated with goat anti-rabbit IgG (Jackson ImmunoResearch Laboratories, Germany), conjugated with 6 nm gold. For analysis we used a Zeiss LIBRA 120 transmission electron microscope (Zeiss, Oberkochen, Germany) operating at 120 kV.

### Transwell membrane assay

*K*. *pneumoniae* was grown in TSB at 37°C to an OD_600_ of 0.2 and washed with 10 mM sodium phosphate (pH = 7.4). 1 x 10^7^ CFU were collected by centrifugation at 7000 rpm, 5 min. The pellet was resuspended in 50 μl AMA buffer (10 mM sodium phosphate, containing 1% TSB). A peptide concentration of 62.5 μmol in a final volume of 50 μl AMA buffer was pipetted into transwell inserts with a semi-permeable polycarbonate membrane (Corning, USA, 3 μm pore size), and incubated 10 min, 30 min or 1 h at 37°C to allow net-formation. Subsequently, 1 x 10^7^ CFU of *K*. *pneumoniae* in 50 μl AMA buffer were added carefully into the inserts. The inserts were placed in a 24-well plate with 400 μl prewarmed TSB and incubated for 1 h, 37°C at 110 rpm to allow bacterial transmigration. After incubation the membrane was removed, 10 μl suspension were plated on blood agar plates and bacterial growth was measured at OD_600_ (BioTek Plate reader Synergy HT, BioTek, USA) after 2 h and % growth of negative control was plotted. Data are presented as mean +/- SEM of at least three independent experiments. The statistic was evaluated by using student’s t-test with ****p < 0.0001.

### Reduction of hBD1ox with thioredoxin

*In vitro* reduction assay performed with 50 μM oxidized hBD1. HBD1ox was incubated with 0.8 mM NADPH (Biomol, Hamburg, Germany), rat TRX reductase (200 nM, IMCO, Stockholm, Sweden), and 3 μM human TRX (Sigma Aldrich) in 100 mM potassium phosphate buffer containing 2 mM EDTA at pH 7.0 for 30 min at 37°C. A control sample was incubated with buffer instead of human TRX. Thereafter, bacteria were exposed to the mixture for 2h at 37°C. Samples were analyzed by scanning electron microscopy. Addition of *E*. *coli* to hBD1ox, human thioredoxin and thioredoxin reductase resulted in formation of nets. Addition of *E*. *coli to* hBD1ox and thioredoxin reductase to in absence of human thioredoxin did not result in net-formation. Magnification bar = 2 μm.

## Supporting information

S1 Fig Scanning electron microscopy of *E*. *coli* with 0.01% acetic acid. *E*. coli treated with acetic acid. Magnification bar = 2 μm. The image shows one representative experiment.
(TIF)

S2 Fig Reduction of hBD1ox with thioredoxin system results in net formation. (A) Addition of thioredoxin mix to *E*. *coli* in presence of hBD1ox resulted in formation of nets. (B) Addition of thioredoxin reductase and hBD1ox to *E*. *coli* in absence of human thioredoxin did not result in net-formation. Magnification bar = 2 μm.
(TIF)

S3 Fig Controls of hBD1 antibodies. (A) Bacteria were incubated with 200 μl 0.01% acetic acid and with antibodies against hBD1red. The secondary antibody is conjugated to gold particles, 6nm (arrows). (B) Bacteria were incubated with 200 μg/ ml hBD1red and with antibodies against hBD1ox. The secondary antibody is conjugated to gold particles, 6 nm. Magnification bar = 0.5 μm.
(TIF)

S4 Fig Transwell membrane assay with different pre-incubations times of peptides for net-formation. Transwell membrane assays were performed as described in the material & methods part. Here we incubated the peptides for (A) 10 min or (B) 30 min at 37°C to allow net-formation. Assays were continued with the described protocol. Net-formation needs a pre-incubation at least of 30 min to build a strong net that hinders bacteria to translocate. Representative blood agar plates are shown. Data are presented as mean +/- SEM of at least three independent experiments. Data are presented as mean +/- SEM of at least three independent experiments. The statistic was calculated by using student’s t-test with (A) *p = 0.0171 and (B) ***p = 0.0001.
(TIF)

S5 Fig Determination of proteolytic activity of human duodenal secretion. Proteolytic activity of human duodenal secretion was assayed by using 2.5% (w/v) azocasein, which was incubated with 2 ml of duodenal secretion from one individual. We used 10 μl of trypsin enzyme solution as positive controls; samples without proteases are the negative controls. Data are presented as mean +/- SEM of two experiments.
(TIF)
