# Supplementary figures and images for: Ubiquitously expressed Human Beta Defensin 1 (hBD1) forms bacteria-entrapping nets in a redox dependent mode of action
Source: PLoS Pathog. 2017 Mar 21;13(3):e1006261. doi: 10.1371/journal.ppat.1006261 (PMC5376342; doi:10.1371/journal.ppat.1006261)

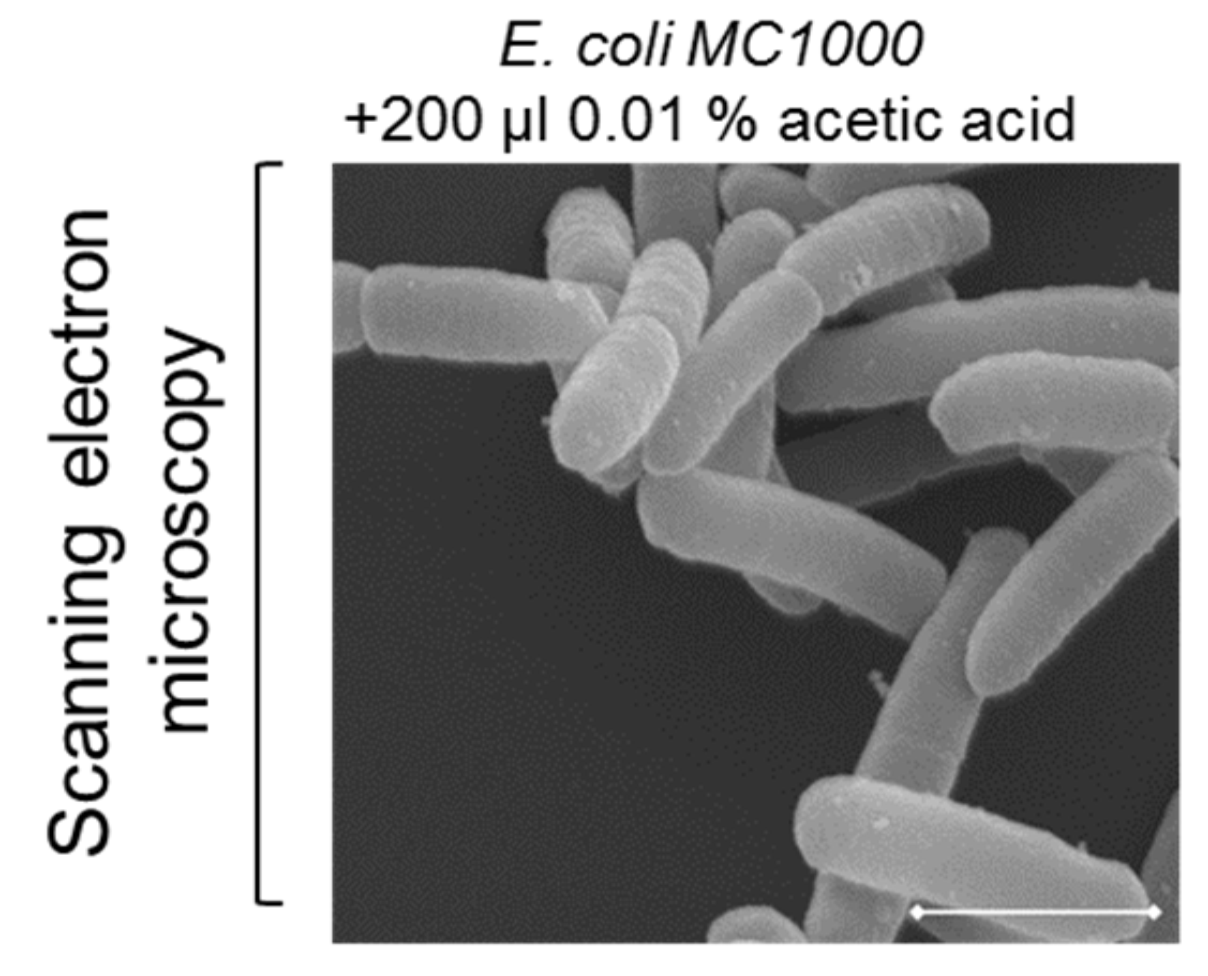

Supplement: S1 Fig — E. coli treated with acetic acid. Magnification bar = 2 μm. The image shows one representative experiment. (TIF) [file ppat.1006261.s001.tif]

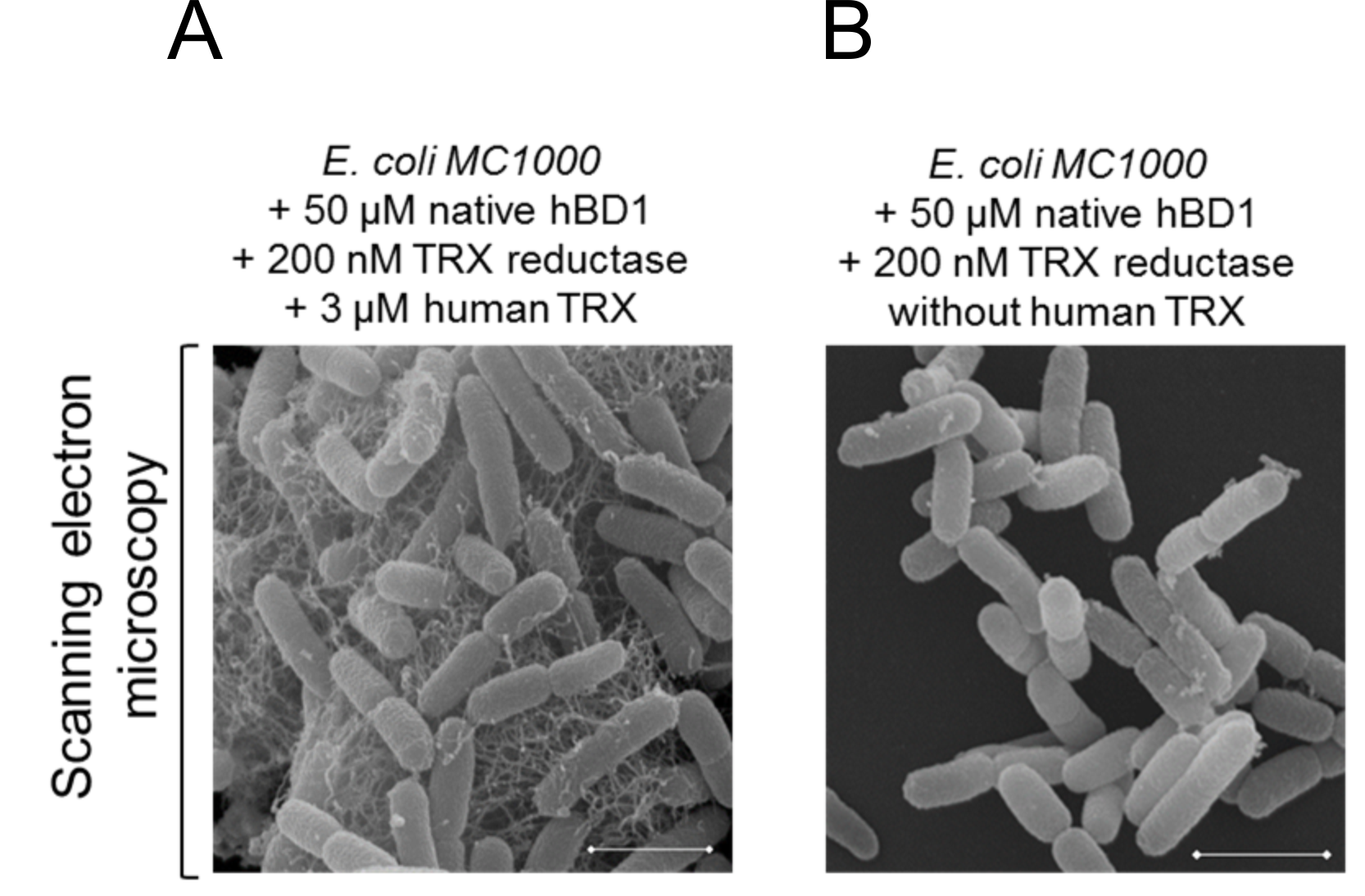

Supplement: S2 Fig — (A) Addition of thioredoxin mix to E. coli in presence of hBD1ox resulted in formation of nets. (B) Addition of thioredoxin reductase and hBD1ox to E. coli in absence of human thioredoxin did not result in net-formation. Magnification bar = 2 μm. (TIF) [file ppat.1006261.s002.tif]

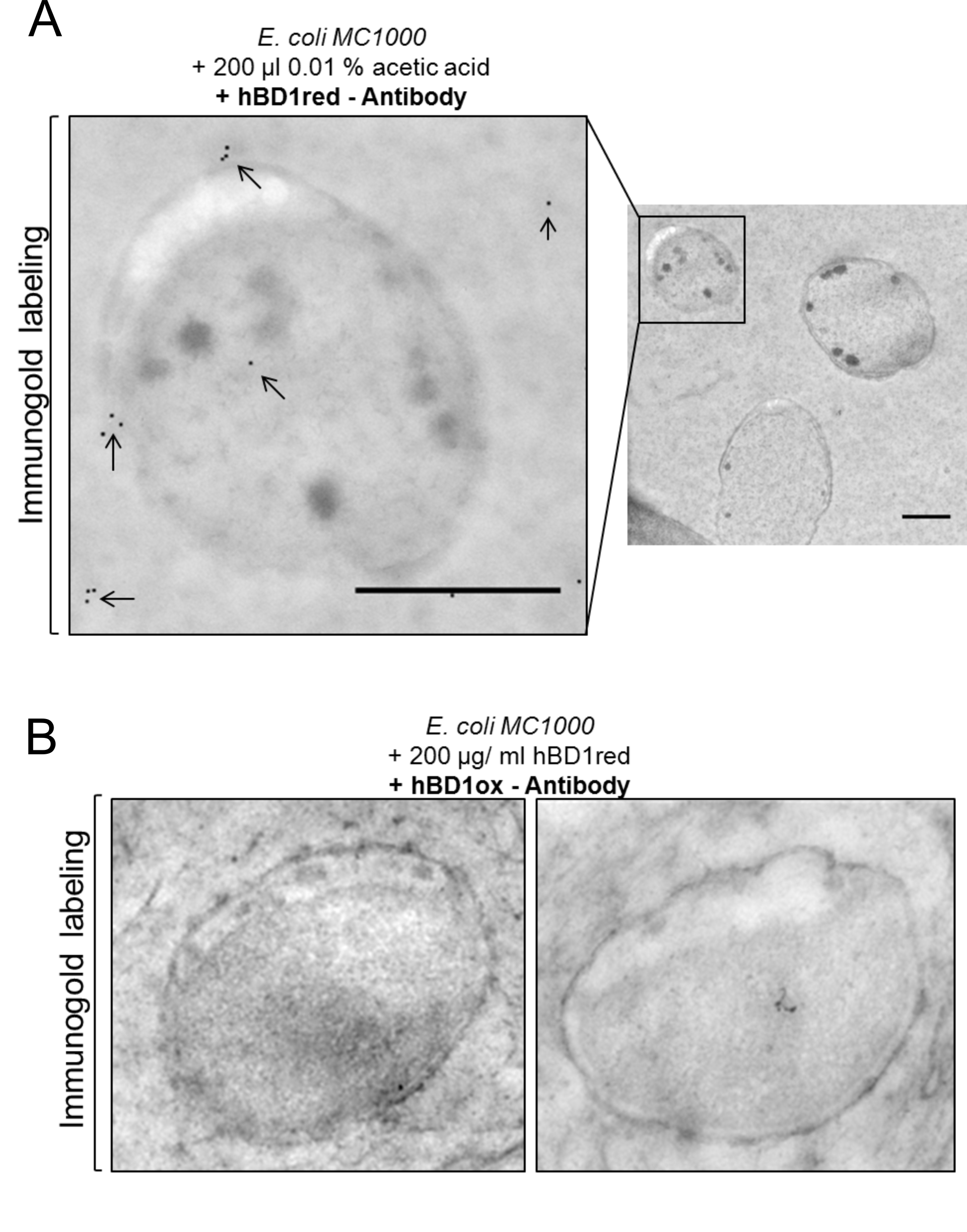

Supplement: S3 Fig — (A) Bacteria were incubated with 200 μl 0.01% acetic acid and with antibodies against hBD1red. The secondary antibody is conjugated to gold particles, 6nm (arrows). (B) Bacteria were incubated with 200 μg/ ml hBD1red and with antibodies against hBD1ox. The secondary antibody is conjugated to gold particles, 6 nm. Magnification bar = 0.5 μm. (TIF) [file ppat.1006261.s003.tif]

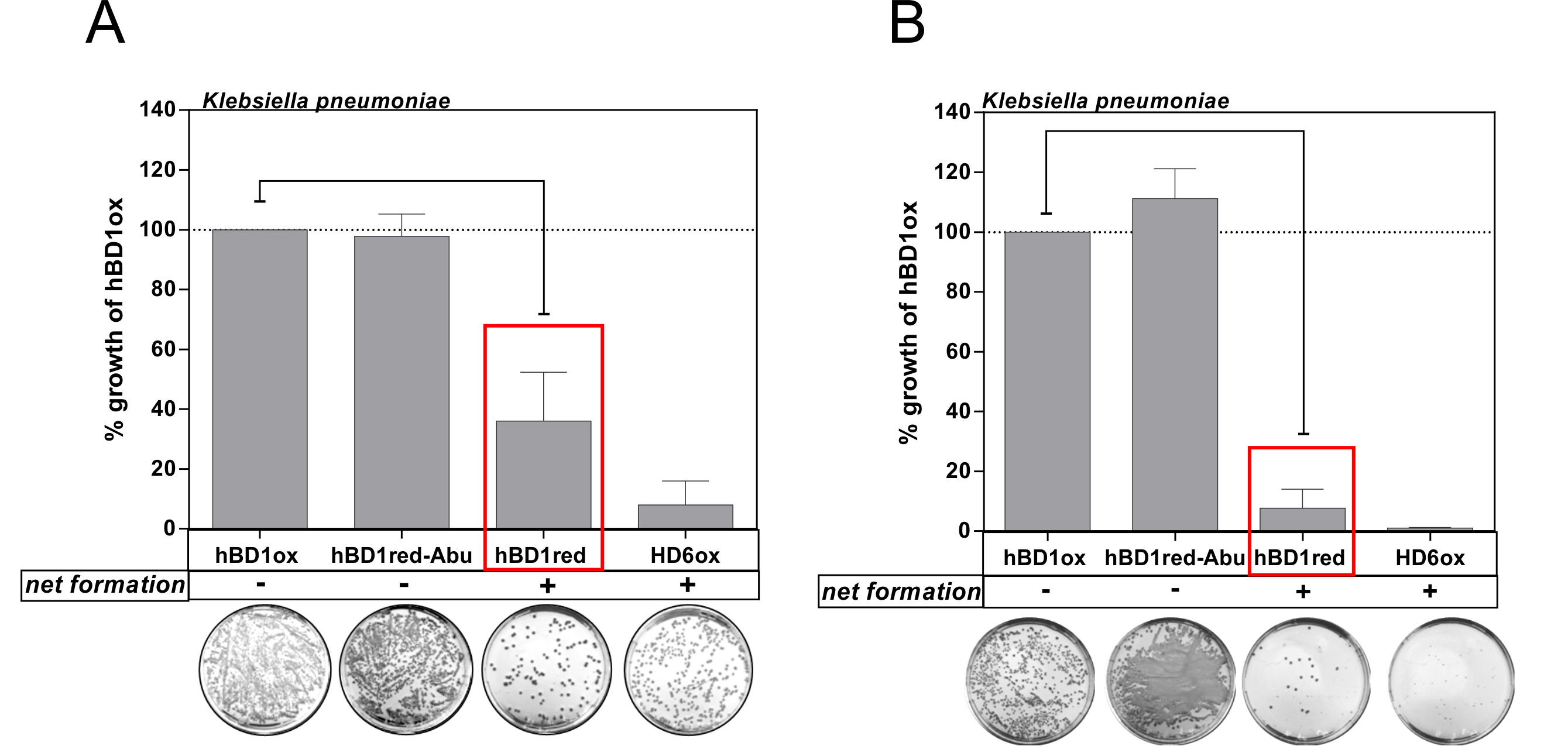

Supplement: S4 Fig — Transwell membrane assays were performed as described in the material & methods part. Here we incubated the peptides for (A) 10 min or (B) 30 min at 37°C to allow net-formation. Assays were continued with the described protocol. Net-formation needs a pre-incubation at least of 30 min to build a strong net that hinders bacteria to translocate. Representative blood agar plates are shown. Data are presented as mean +/- SEM of at least three independent experiments. Data are presented as mean +/- SEM of at least three independent experiments. The statistic was calculated by using student’s t-test with (A) *p = 0.0171 and (B) ***p = 0.0001. (TIF) [file ppat.1006261.s004.tif]

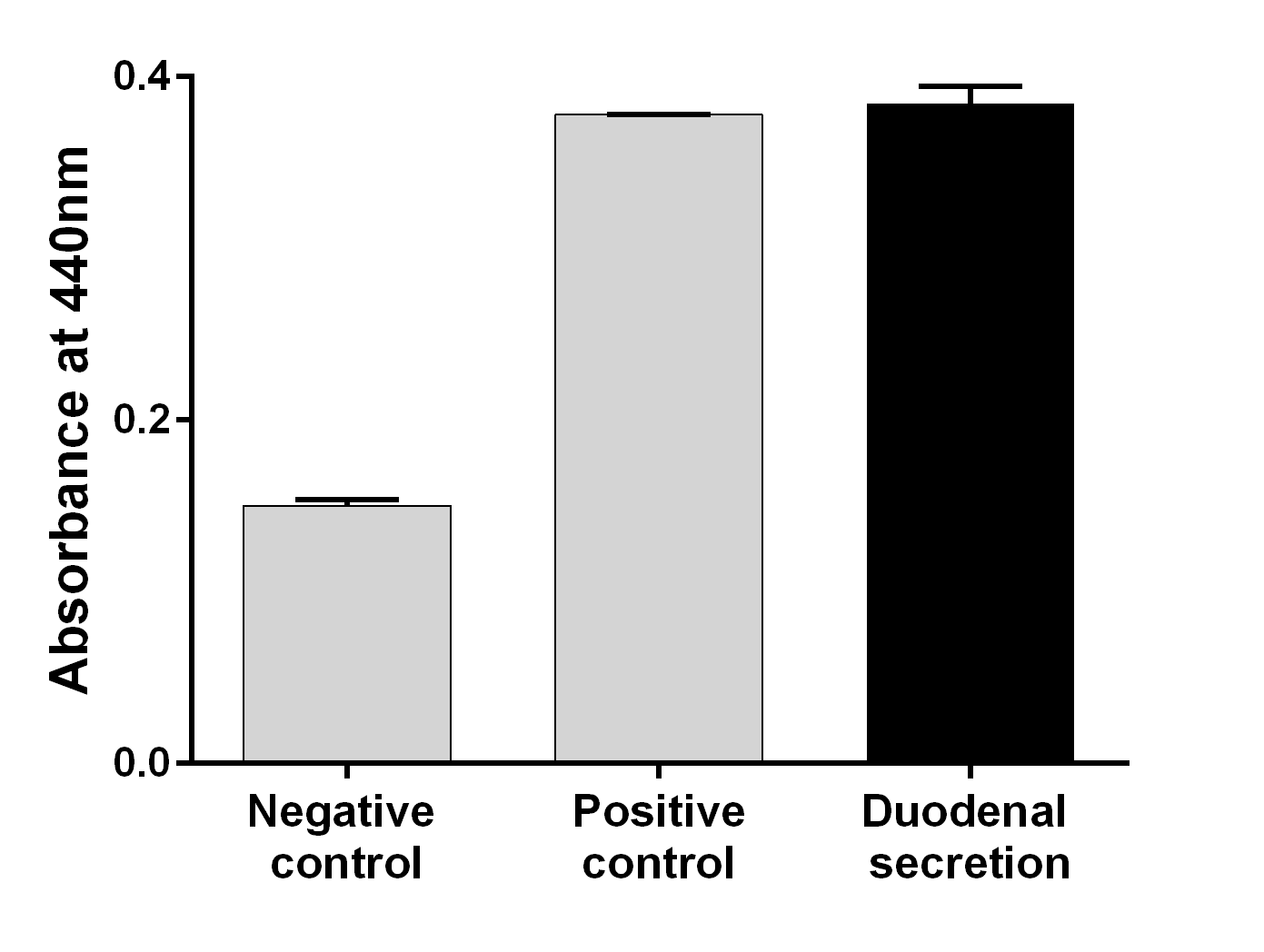

Supplement: S5 Fig — Proteolytic activity of human duodenal secretion was assayed by using 2.5% (w/v) azocasein, which was incubated with 2 ml of duodenal secretion from one individual. We used 10 μl of trypsin enzyme solution as positive controls; samples without proteases are the negative controls. Data are presented as mean +/- SEM of two experiments. (TIF) [file ppat.1006261.s005.tif]
